# Increasingly dependent on habit? A study on the electrophysiological mechanisms of goal–directed and habitual control in internet gaming disorder

**DOI:** 10.1556/2006.2024.00084

**Published:** 2025-05-15

**Authors:** Xuemei Gao, Jiangmiao Lu, Yating Huang, Ling Wang

**Affiliations:** 1 Psychological Research and Consultation Center, Southwest Jiaotong University, No. 999 Xian Road, Chengdu, Sichuan, China; 2 Faculty of Psychology, Southwest University, Chongqing, China; 3 Key Laboratory of Cognition and Personality, Ministry of Education, Southwest University, Chongqing, China; 4 Faculty of Mathematics and Statistics, Southwest University, Chongqing, China; 5 School of General Education, Chongqing Vocational Institute of Engineering, Chongqing, China

**Keywords:** internet gaming disorder, goal–directed, habitual, instrumental learning, electroencephalography

## Abstract

**Background:**

Public health issues arising from excessive online gaming have garnered significant research interest. Existing studies indicate that, within the framework of the dual-systems theory, the equilibrium between the goal–directed and habitual control systems is disrupted in patients with Internet gaming disorder (IGD). Nevertheless, the understanding of how this imbalance manifests within the brain is limited. This study aims to investigate real-time brain activity in individuals with IGD during the activation of both the goal–directed and habitual systems using electrophysiological techniques.

**Methods:**

Twenty-four individuals with IGD and twenty-three matched recreational game users (RGUs) underwent electroencephalography (EEG) data collection while completing an outcome devaluation task. Differences between the two groups at the Fz, Cz, and Pz electrodes were compared using repeated measures ANOVA.

**Results:**

The behavioral results revealed that the RGU group exhibited higher accuracy than the IGD group during the learning phase (*t*(45) = −3.08, *p* < 0.001, *η*^2^ = 0.42). During the slip-of-action test, the IGD group made more action-slip responses for devalued outcomes than the RGU group (*F*_(1,45)_ = 6.22, *p* = 0.016, *η*^2^ = 0.12). The EEG experiment results indicated that, upon stimulus presentation during the slip-of-action test, the IGD group had significantly more negative average amplitudes at the Fz and Cz electrodes compared with the RGUs (−7.26 ± 6.28 μV; −5.18 ± 5.49 μV; *F*_(1,40)_ = 5.54, *p* = 0.024, *η*^2^ = 0.12; *F*_(1,40)_ = 4.92, *p* = 0.032, *η*^2^ = 0.11). Concurrently, the single-group analysis based on RGU revealed that habitual control appears to be associated with greater N2 and P3 amplitudes during the stimulus-locked phase.

**Conclusions:**

The goal–directed system of individuals with IGD is impaired, manifesting in the increased cognitive resources required to activate the goal–directed system when they need to disrupt habitual responses. This suggests that the imbalance due to IGD within the dual systems may originate from an impaired goal–directed system rather than the overactivation of the habitual system.

## Introduction

The Internet is a paramount tool for information exchange in contemporary society, which promotes widespread dissemination and sharing of information, altering people's lifestyles. However, it has precipitated a series of health-related concerns, such as problematic pornography and social networking use, problematic shopping, and excessive online gaming (Brand, 2022). The issues arising from excessive online gaming in schools, families, and society have attracted significant attention. Internet gaming disorder (IGD), as classified in DSM-5 and ICD-11, typically refers to persistent and recurrent engagement in online or offline games, resulting in significant impairment or distress within a period of 12 months (Rehbein, Kliem, Baier, Mößle, & Petry, 2015; WHO, 2017). Previous studies have indicated that globally, the prevalence of IGD is approximately 3.05%, with notable regional disparities (Stevens, Dorstyn, Delfabbro, & King, 2021). In China, as of 2022, the number of game users has reached 664 million, nearly exceeding half of the total population of China (*2022 China Game Industry Report, 2023*). Given the substantial number of game users, the effects of IGD must not be underestimated. Therefore, the investigation of IGD and its underlying mechanisms is pivotal, contributing to a deeper comprehension of IGD complexities and offering more targeted intervention and prevention strategies to mitigate the adverse effects of IGD on individuals and society.

The dual-system theory (Dickinson & Balleine, 1993) posits that individual behavior can be divided into two processes: one representing goal–directed actions and the association between actions and outcomes, and the other representing habits as the link between stimuli and responses (Everitt & Robbins, 2016). Moreover, the habitual control and goal–directed systems are relatively independent yet mutually influential (Wood, Mazar, & Neal, 2022). Typically, the goal–directed and habitual systems collaboratively regulate human behaviors and exist in a delicate balance (Dickinson & Balleine, 1993). However, when individuals repetitively engage in rewarded behavior in a stable environment (e.g., continual exposure to games), the balance between the habitual control and goal–directed systems is disrupted, leading to a tendency for behavior to incline toward habitual control (Verplanken & Orbell, 2022; Wood & Ruenger, 2016). Patients diagnosed with IGD demonstrate a heightened dependence on habitual regulation, which leads to exacerbated challenges when attempting to disrupt well-entrenched behavioral patterns (Dong et al., 2021; Zhou et al., 2021). Nevertheless, the precise neurophysiological mechanisms that underlie this transition continue to elude researchers.

Previous research has frequently used the outcome devaluation task to examine both goal–directed and habitual responses (de Wit, Niry, Wariyar, Aitken, & Dickinson, 2007; Dong et al., 2021; Ersche et al., 2016; Luijten et al., 2020; Sjoerds et al., 2013; Zhou et al., 2021). Given that habit formation relies on the core principle of repeated reward responses, the initial phase of the outcome devaluation task generally involves establishing instrumental learning responses (stimulus–response–outcome). This is then followed by outcome devaluation, achieved either through satiety (Everitt, 2014) or by instructing participants to devalue the currency. The subsequent test phase evaluates whether participants can modify their behavior in response to changes in the outcome value: habitual control responses remain persistent toward these devalued outcomes. The outcome devaluation paradigm has demonstrated an advantage of habitual control over goal–directed behavior in obsessive-compulsive disorder (Gillan et al., 2015), eating disorders (Wang et al., 2023), and other maladaptive disorders associated with addiction (Ersche et al., 2016; Luijten et al., 2020; Sjoerds et al., 2013). Despite some studies questioning the validity of using the outcome devaluation paradigm to study habits—arguing that its definition (insensitivity to changes in outcome value) is narrower than the habitual behaviors encountered in the real world (Watson & de Wit, 2018; Watson, O'Callaghan, Perkes, Bradfield, & Turner, 2022)—the outcome devaluation paradigm remains a productive research avenue (Donamayor et al., 2022; Wood et al., 2022).

From an evolutionary standpoint, habits are not inherently pathological; rather, they represent an efficient behavioral pattern. Nonetheless, in the context of addictive and overused behaviors, they may result in numerous adverse outcomes for individuals (Robbins & Everitt, 1999). According to the incentive sensitization theory of addiction, individuals may transition from initial recreational use to compulsive addictive behavior, possibly experiencing a shift from “liking” to “wanting” (Berridge & Robinson, 2016) or from “feeling better” to “must do” (Brand, 2022). This progression suggests that habit formation could serve as the fundamental basis for maladaptive or addictive behaviors (Everitt & Robbins, 2016).

Within the context of drug addiction, maladaptive habits, which are prevalent in human psychiatric disorders, have been demonstrated to progressively seize control of the brain, resulting in reduced sensitivity to devalued outcomes and heightened dependence on habitual behaviors (Ersche et al., 2016, 2021; Sjoerds et al., 2013). Compared with healthy individuals, individuals with cocaine addiction demonstrate compromised learning abilities in appetitive instrumental learning tasks, along with heightened reactivity toward devalued outcomes during subsequent outcome devaluation tasks (Ersche et al., 2016). Likewise, individuals struggling with alcohol dependence demonstrate heightened inclinations toward habits and reduced activity in brain regions linked to goal–directed actions (e.g., the ventromedial prefrontal cortex and anterior cingulate cortex), while showing amplified activation in brain areas associated with habit formation (e.g., the dorsal striatum) on neuroimaging scans (Sjoerds et al., 2013). Furthermore, individuals with nicotine addiction and obsessive-compulsive disorder demonstrate transitions between the dual systems similarly (Gillan et al., 2015; Luijten et al., 2020). Additionally, a propensity toward habitual shifts manifests in behavioral addictions, as evidenced by studies indicating that individuals with Internet addiction tend to develop stimulus-driven habits and exhibit greater difficulty in disengaging from these entrenched patterns (Zhou, Wang, Zhang, Li, & Nie, 2018). In the context of IGD, both task-based and resting-state functional magnetic resonance imaging (fMRI) methods and behavioral observations reveal an imbalance between habitual and goal–directed systems; on fMRI, this is evidenced by aberrant activations within the dorsal striatum, ventral striatum, and thalamocortical pathways (Dong et al., 2021; Zhou et al., 2021), which are critical neural substrates implicated in the regulation of habitual behaviors and goal–directed processes (Gremel & Costa, 2013; O'Hare et al., 2016; Yin, Knowlton, & Balleine, 2004). Although the disparities between dual systems in IGD have garnered increasing research interest, prior investigations have predominantly concentrated on behavioral and fMRI modalities, with the volume of research remaining insufficient. Hence, more comprehensive and rigorous evidence is required to elucidate, from a dual-system standpoint, the gradual entrapment of individuals with IGD within the “habit” trap.

To investigate the neural activity associated with breaking established habits over time in individuals with IGD compared with recreational game users (RGUs), this study employed event-related potential techniques and the outcome devaluation task used in previous research efforts (Ersche et al., 2016; Luijten et al., 2020). At the electrophysiological level, given that the slip-of-action test in the outcome devaluation task bears resemblance to a traditional go/no-go task, both goal–directed and habitual control systems can be observed in the N2 and P3 components during stimulus presentation. The N2 component is generally characterized by a negative deflection occurring between 200 and 400 ms post-stimulus presentation, reflecting attentional allocation and inhibitory control in response to task-relevant stimuli (Bekker, Kenemans, & Verbaten, 2005; Botvinick, Braver, Barch, Carter, & Cohen, 2001). The P3 component typically manifests as a positive deflection between 300 and 500 ms after stimulus presentation. The cognitive functions associated with the P3 component are analogous to those of the N2 component, encompassing post-response monitoring and the evaluation of inhibitory control (Kok, Ramautar, De Ruiter, Band, & Ridderinkhof, 2004; Picton, 1992; Pritchard, 1981). Findings from a recent meta-analysis suggest that compared with healthy controls, individuals with IGD exhibit compromised inhibitory control, as evidenced by larger amplitudes in the N2 and P3 components (Yu, Abdullah, & Mansor, 2024). Consequently, we hypothesize that individuals with IGD experience greater difficulty in activating goal-oriented systems, which may result in increased N2 and P3 amplitudes. Conversely, we expect that during habitual control, participants with IGD may struggle with recognizing errors, leading to reduced N2 and P3 amplitudes.

When individuals rely on habitual control, particularly in the context of an action slip, the event-related negativity (ERN) and error positivity (P_E_) components are of particular interest, given that action errors fundamentally represent such responses. The ERN is characterized by a central negative deflection, peaking between 50 and 80 ms after erroneous responses, and is generally considered to reflect the monitoring of conflict between response tendencies and expected errors. Subsequent behavioral adjustments and response conflicts can amplify the ERN (Gehring & Fencsik, 2001; Hajcak, McDonald, & Simons, 2004; Holroyd & Coles, 2002). The P_E_ component, which follows the ERN, manifests as a positive deflection over the parietal cortex, peaking between 200 and 500 ms, and is broadly regarded as reflecting error awareness and corrective responses (Boldt & Yeung, 2015; Nieuwenhuis, Ridderinkhof, Blow, Band, & Kok, 2001). We hypothesize that individuals with IGD will display reduced ERN and P_E_ amplitudes following an action slip.

Furthermore, we are interested in examining the differences in feedback-related negativity (FRN) between the two groups during the learning phase. FRN is characterized by a negative deflection, peaking 200–300 ms after feedback stimuli. FRN is believed to reflect neural responses to outcomes that are worse than expected (Bellebaum, Polezzi, & Daum, 2010) or to the evaluative significance of feedback in relation to the outcome (Gehring & Willoughby, 2002). IGD patients are reported to exhibit insensitivity to negative rewards (Cho, Nah, Park, & Han, 2022; Zhang, Hu, Wang, Wang, & Dong, 2020). Consequently, we hypothesize that participants with IGD will demonstrate reduced FRN activation in response to negative feedback during the instrumental learning phase. In addition to comparing the electrophysiological differences between the IGD and RGU groups, we also conducted a focused investigation into the characteristics of habitual responses based on electrophysiological data from healthy participants.

In addition to comparing the electrophysiological differences between IGD and RGU, we specifically investigated the characteristics of habitual responses based on the electrophysiological data from healthy participants. Previous studies focusing on healthy participants have indicated that goal-directed behavior is associated with lower N2 amplitudes following stimulus presentation and higher ERN amplitudes after responses (Yousuf, Heldmann, Munte, & Donamayor, 2019), reflecting the differences between the two types of responses (habitual and goal-directed). Therefore, we also included RGUs as subjects instead of IGD players. Given that IGD players may experience an imbalance between habitual control and goal-directed behavior, as revealed in previous studies (Dong et al., 2021; Zhou et al., 2021), the electrophysiological indicators of the dual systems in IGD players may be atypical, potentially exaggerating or diminishing the differences in electrophysiological measures between habitual and goal-directed responses. Therefore, at this stage, we selected RGU players for our investigation.

In summary, our study represents the first exploration of brain responses in IGD individuals and their comparison with those in RGUs on a high-resolution temporal scale, when engaging in habitual versus goal–directed behaviors. Except this, we also delineate the electrophysiological components associated with goal-oriented and habitual performance using the result devaluation paradigm.

## Method

### Participants

This study was conducted in China between April and September 2023, and all participants were Chinese college students from Southwest University. The participants were recruited through the Questionnaire Star platform, which is an online questionnaire distribution and collection program (questionnaires are shared on the Internet by generating links). Prior to their involvement in the experiment, all participants completed a hard copy of the informed consent form and provided background data, including age, gender, handedness, education, weekly gaming hours, and the proportion of gaming hours to Internet usage. Individuals previously diagnosed with mental disorders other than IGD, such as depression, anxiety, schizophrenia, and substance use disorders, were excluded from both the experimental and control groups.

Sample size calculations were performed using G*Power 3.1. The analysis, which utilized a repeated-measures ANOVA for interactions with an effect size of (*f* = 0.25) and a significance level of (*α* = 0.05), indicated that a minimum of 44 participants is required to achieve 95% power for our primary analyses. The initial screening was conducted using Young's (1989) Internet addiction test (IAT) for IGD and the DSM-5 nine-item IGD diagnostic scale (APA, 2013). A total of 30 participants with IGD and 28 RGUs met the criteria. These participants completed additional self-report measures. In the IGD group, one participant with alcohol dependence, three with nicotine dependence, one previously diagnosed with depression, and one who did not complete the post-test written assessment were excluded. In the RGU group, one participant with alcohol dependence, two with nicotine dependence, and two who did not complete the written assessment were excluded.

Finally, 24 IGD participants (14 men, 10 women) who met five or more of the nine DSM-5 IGD diagnostic criteria (IAT score: 74.71 ± 12.99) and 23 RGUs (13 men, 10 women) who met fewer than five of the DSM-5 criteria (IAT score: 39.78 ± 7.11) participated in this study. All participants played games for more than 10 h per week, had a gaming history of more than one year, did not have major physical illnesses, were not diagnosed with depression or other mental disorders in the past, had an Alcohol Use Disorder Identification Test score of less than 8, and a Fagerstrom Test for Nicotine Dependence score of less than 3. The demographic information of all participants is shown in Table 1.

**Table 1.** Demographic information and group differences

	IGD (*n* = 24)	RGU (*n* = 23)	t/χ2	*p*
Sex	Male = 14	Male = 13	−0.12	0.903
	Female = 10	Female = 10		
Age (Mean ± SD)	20.88 ± 2.21	21.26 ± 2.38	−0.58	0.567
Education (Mean ± SD)	15.08 ± 1.64	14.65 ± 2.25	0.75	0.455
DSM (Mean ± SD)	7.45 ± 1.41	1.57 ± 1.04	16.24	0.000
IAT (Mean ± SD)	74.71 ± 12.99	39.78 ± 7.11	11.50	0.000
SPAN (Mean ± SD)	5.42 ± 1.10	5.76 ± 1.01	−1.12	0.270
OCI (Mean ± SD)	24.67 ± 13.40	21.57 ± 9.62	0.91	0.369
Aggressiveness (Mean ± SD)	43.92 ± 19.98	39.78 ± 15.92	0.78	0.438
Impulsion (Mean ± SD)	18.41 ± 3.68	18.00 ± 4.20	0.36	0.719
Alcohol (Mean ± SD)	2.58 ± 2.30	1.83 ± 2.01	1.20	0.237
Nicotine (Mean ± SD)	0.75 ± 1.22	0.52 ± 1.08	0.68	0.502

IGD: Internet gaming disorder; RGU: Recreational game user; IAT: Internet addiction test; DSM-5: IGD Diagnostic scale from Diagnostic and Statistical manual of Mental Disorders-5; SPAN: Working memory span; OCI: Degree of obsessive-compulsive disorder.

### Tools

#### Internet gaming disorder

During the screening process, all participants completed Young's IAT specifically designed for IGD (Moon et al., 2018). The IAT questionnaire was originally devised by Young to address issues of Internet addiction. Subsequently, researchers adapted it to assess IGD (substituting “Internet” with “online gaming” in each item); the revised questionnaire has demonstrated robust construct validity and found widespread utility in IGD screening initiatives (Dong et al., 2021; Zhou et al., 2021). The IAT questionnaire employs a five-point scale for each item, ranging from 1, signifying “rarely,” to 5, signifying “always,” primarily addressing aspects of psychological dependency, compulsive usage, work, academic pursuits, and familial obligations, comprising a total of 20 items with scores ranging from 20 to 100 (Young, 1989). A score of more than 50 indicates that an individual is at high risk for IGD.

Furthermore, participants completed the 9-item IGD diagnostic scale from DSM-5 (Petry et al., 2014), which includes criteria such as gaming obsession, withdrawal symptoms, and increased tolerance. Responses were recorded as either “Yes” or “No,” with meeting five or more of the nine criteria indicating a higher risk of IGD. The concurrent use of IAT and DSM-5 in research on IGD is considered an effective means of IGD screening (Ko et al., 2014).

#### Additional control variables

In addition, participants completed the Brief Barratt Impulsiveness Scale (Steinberg, Sharp, Stanford, & Tharp, 2013), Alcohol Use Disorder Identification Test (Saunders, Aasland, Babor, de la Fuente, & Grant, 1993), Fagerstrom Test for Nicotine Dependence (Heatherton, Kozlowski, Frecker, & Fagerström, 1991), Obsessive-Compulsive Inventory-Revised (Foa et al., 2002), the Depression–Anxiety–Stress Scales (Lovibond & Lovibond, 1995), and the Chinese College Student Version of the Buss-Perry Aggression Questionnaire (Buss & Perry, 1992; Xianyun et al., 2011). These variables were subsequently used as covariates, and all questionnaires and scales were completed via self-report by the participants.

### Stimuli and procedure

#### Procedure

All experimental procedures were programmed using E-Prime 2.0, displayed on a 17-inch color CRT monitor, with a resolution of 1,280 × 768 and a refresh rate of 60 Hz. Two undergraduate students, who were not psychology majors, were recruited online to serve as experimenters. They underwent three days of training on the use of the equipment and were unaware of the purpose of the experiment prior to their involvement.

The experiment was conducted in two phases. Phase One was a working memory capacity test, which employed the Corsi block-tapping test (Corsi, 1973) to assess participants' working memory capacity. In this test, a 5 × 5 grid of blank squares was displayed on the screen, followed by a sequence of green squares which participants were required to click in the order of their appearance.

**Fig. 1.**
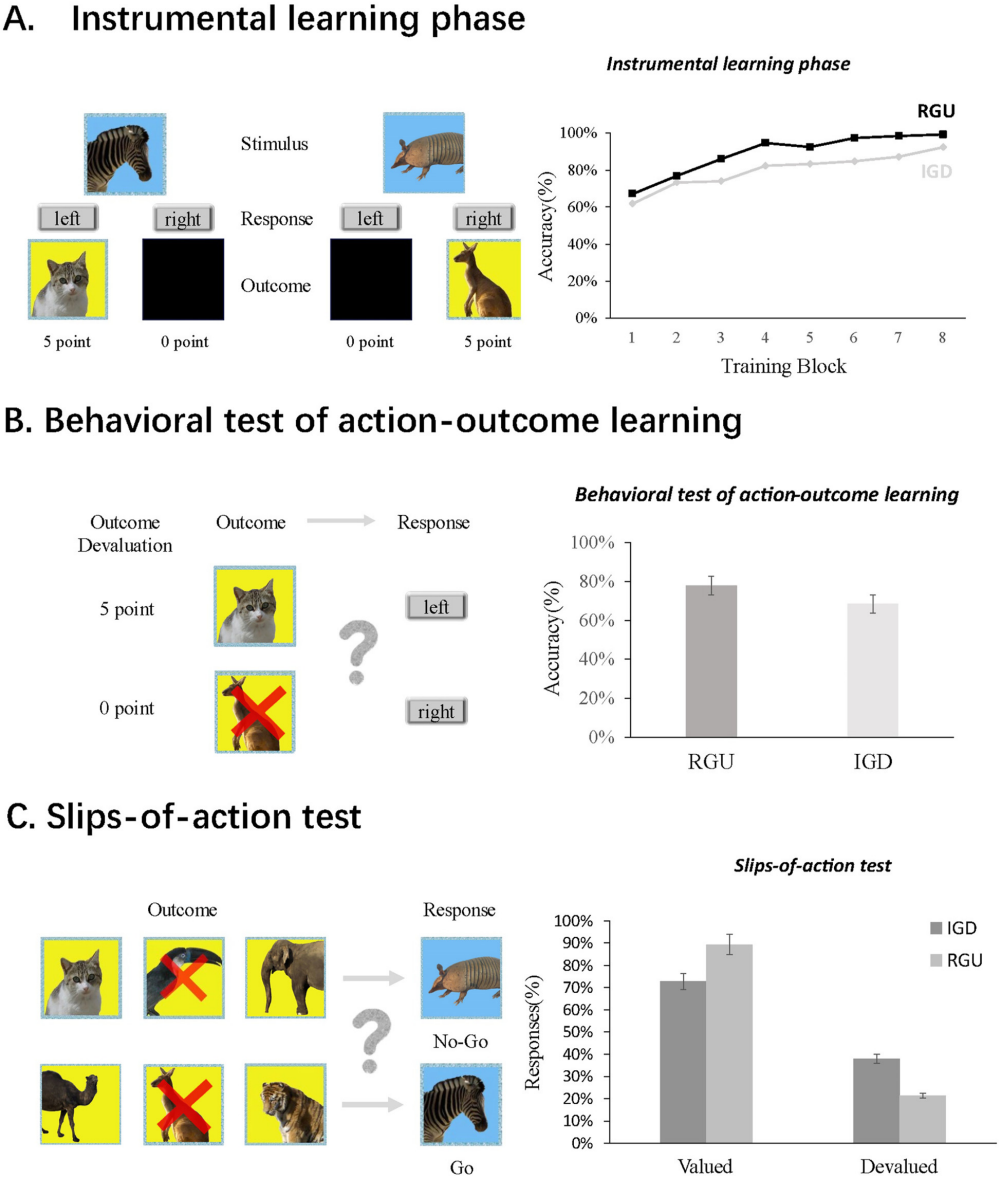
The outcome devaluation task. The figure shows the process of the outcome devaluation task, which was developed to differentiate between goal–directed control and habitual learning. (A) During the instrumental learning phase, participants learn stimulus–response–outcome associations, which is stimulated by reward points. (B) In the behavioral test of action-outcome learning, one of the two displayed outcomes is devalued (indicated by a red cross in the image). The participants are instructed to respond with the correct response that is associated with the outcome. (C) In the slips-of-action test, all outcomes are displayed to the participants. Two of the outcomes are devalued. After outcome presentation, the stimuli appear one by one on the screen. Participants are required to respond with the correct learning response, unless the outcomes related to the stimulus are devalued. At this point, participants with strong habitual tendencies will automatically respond to stimuli with learned responses, regardless of the value of the relevant outcomes. IGD: Internet gaming disorder; RGU: recreational game user

Phase Two involved the outcome devaluation tasks, which comprised three components (instrumental learning phase, action–outcome test, slip-of-action test), aligning with the paradigms used in prior studies on cocaine and smoking addictions (Ersche et al., 2016; Luijten et al., 2020). During the instrumental learning phase (see Fig. 1A), participants had to learn the associations between stimuli, responses, and outcomes to earn reward points. Before the experiment, the participants were informed that points would be converted into additional compensation at the end of the experiment. In the experiment, participants initiated the task by pressing the spacebar after reading the instructions, which were presented without a time limit. The fixation cross was presented for 500 ms. The duration of both the instructions and fixation cross display was consistent across all stages. Following the presentation of stimuli (represented by animal images with a blue background in the task, and each stimuli was displayed for 2,000 ms), participants were instructed to press either the left or right key on the keyboard. If correct, they received an outcome corresponding to the stimulus (indicated by animal images with a yellow background) and earned reward points. Incorrect responses resulted in an error prompt and no reward points, while both correct outcomes and error prompt were presented for 2,000 ms. The mapping between stimuli, responses, and outcomes is fixed (for instance, the blue zebra corresponds to the left key and the yellow cat, while the blue armadillo corresponds to the right key and the yellow kangaroo). There were six different stimuli and six different outcomes, each pair used twice per block. Initially, participants were unaware of the correct match and had to engage in trial-and-error learning. The learning phase comprised a total of eight blocks, with each block consisting of twelve trials, and an inter-trial interval of 1,500 ms.

During the action–outcome test (see Fig. 1B), participants were tested on their learning of the association between the outcomes and responses from the previous phase. Two randomly selected outcomes (corresponding to the left and right keys) from the six outcome images were simultaneously displayed on the screen for 2,000 ms, and participants were informed that one outcome was no longer valuable (indicated by a red × on the image). To earn points, they were instructed to perform responses associated with the outcome that remained valuable. For example, when a yellow cat and a yellow kangaroo appeared simultaneously, with the kangaroo devalued, participants were instructed to respond to the cat. No feedback was provided during this testing phase to prevent further learning. Each trial (a total of 36) was followed by an inter-trial interval of 1,500 ms. Moreover, the probability of devaluation for each outcome images were equal across all participants, set at 1/6.

In the slip-of-action test, the six outcomes presented during the learning phase were displayed simultaneously on the screen for 10 s, with two outcomes indicated as no longer valuable (represented by a red ×). The same outcomes were devaluated as in the action-outcome test. Participants were instructed that responding to stimuli associated with devalued outcomes would result in point deductions; therefore, they needed to selectively respond to stimuli associated with outcomes from which they could earn points. Following this devaluation procedure, stimuli were presented to participants in a continuous, randomized sequence, with each stimulus displayed for 2,000 ms and an inter-trial interval of 1,500 ms. This phase comprised a total of nine blocks, with each block consisting of twelve trials. In this phase, three types of participant responses were of interest and were subsequently analyzed in the data statistics: (a) following the presentation of stimuli associated with non-devalued outcomes, participants made correct responses; (b) after the presentation of stimuli associated with devalued outcomes, participants correctly inhibited responses and did not react, which typically indicates the functioning of the goal–directed system; and (c) following the presentation of stimuli associated with devalued outcomes, participants failed to inhibit responses and continued to react, which is considered indicative of the activation of the habitual system.

Upon completion of the task, all participants were immediately asked to complete a paper-based test. In this test, participants were required to recall the matching between the presented stimulus images, key presses, and outcome images. Participants whose accuracy did not reach 100% were excluded.

#### Electroencephalography data acquisition and preprocessing

Electroencephalography (EEG) data were recorded using a 64-electrode cap extended from the international 10–20 system (Nuwer et al., 1998) and the ERPs system from Neuroscan (Australia), with a Neuroscan SynAmps2 64-channel amplifier. EEG signals were sampled at 500 Hz with a resolution of 0.1 mV. The ground electrode was located in the AFz, while the reference was on no position of the (extended) 10–20 system. Electrodes above and below the left eye and at the outer canthus of each eye were used to record vertical and horizontal eye movements. Impedances were kept below 10 kΩ.

EEG data preprocessing was conducted using the EEGLAB and ERPLAB extension toolboxes in MATLAB 2022b, with bilateral mastoid electrodes (M1 and M2) used for offline re-referencing. High-pass filtering was set at 0.1 Hz and low-pass filtering at 30 Hz. Interpolation with bad channels was used for damaged channels, followed by independent component analysis to correct eye, ECG, and muscle artifacts (Jung et al., 2000). Data were then segmented from −500 to 1,200 ms based on events of interest. Segments with EEG wave amplitudes exceeding ±100 μV were rejected, and participant data with more than 25% of segments removed were excluded from statistical analysis (data of five participants were removed, two were IGD and three were RGU), followed by averaging for each condition. Baseline correction was applied to the stimulus-locked data from −200 to 0 ms and to the response-locked data from −400 to −200 ms (Yousuf et al., 2019).

### Behavioral and EEG data analysis

Behavioral data were analyzed using SPSS 26.0. For the instrumental learning phase, we initially calculated the accuracy of subjects in each block. Subsequently, time (blocks 1–8) was treated as a within-subjects variable, and the group as a between-subjects variable in a repeated measures ANOVA (rmANOVA). Differences between the two groups in the final block were analyzed using an independent samples *t*-test. In the action–outcome test, differences in accuracy between the two groups across 36 trials were analyzed using an independent samples *t*-test. During the slip-of-action test, a 2 (group: IGD, RGU) × 2 (outcome type: devalued, non-devalued) rmANOVA was conducted with the group as a between-subjects variable and outcome type as a within-subjects variable. If the sphericity assumption was violated, Greenhouse–Geisser corrections were applied to adjust the within-subject degrees of freedom, with post hoc comparisons conducted using the least significant difference corrections.

The average amplitude of midline electrodes (Fz, Cz, Pz) within the time window of interest was used to assess statistical differences between conditions. This time window was based on earlier publications (Donkers & van Boxtel, 2004; Gajewski & Falkenstein, 2013; Huster, Enriquez-Geppert, Lavallee, Falkenstein, & Herrmann, 2013; Luus, Snellenberg, & Liotti, 2007; Nunez-Estupinan, Berticelli, de Almeida, & Gauer, 2022; Yau, Potenza, Mayes, & Crowley, 2015) and validated through visual inspection of the overall average waveform. In the instrumental learning phase, the time window for the FRN was 220–320 ms. To examine differences in the maximum negativity between the IGD and RGU groups across electrode sites (Fz, Cz, Pz) and feedback valence (positive and negative) within this time window, rmANOVA was employed. In the slip-of-action test, the N200 and P300 components were analyzed within the time windows of 200–280 ms and 350–500 ms, respectively, using rmANOVA. This analysis included the following factors: group (IGD and RGU), subsequent responses (response to valuable outcomes, inhibition of devalued outcomes, and slip of action), and electrode sites (Fz, Cz, Pz). For response-related effects, average and peak amplitudes of ERN and P_E_ were measured in the 20–80 ms and 400–500 ms time windows, with independent samples *t*-tests employed to compare ERN and P_E_ differences between the two groups following action slips. Additionally, we compared the electrophysiological differences between habit and goal conditions, using rmANOVA to compare RGUs across electrode sites (Fz, Cz, Pz) and response types (inhibition of devalued outcomes, slip of action). A paired sample *t*-test was used to compare the differences between the slip-of-action test and correct responses to valuable outcomes in ERN and P_E_. If Mauchly's sphericity assumption was violated, Greenhouse–Geisser corrections were applied to adjust within-subjects degrees of freedom, and paired comparisons were conducted using the least significant difference corrections to explore significant main effects and interactions. Detailed data can be found in the supplementary Table.

### Ethics

The experiment complied with the ethical guidelines of the World Medical Association (Helsinki Declaration) and was approved by the Ethics Committee of the Department of Psychology, Southwest University.

## Results

### Behavioral results

#### Stage 1: Instrumental learning phase

For the instrumental learning phase (Fig. 1A), a 2 (group: IGD, RGU) × 8 (block: 1–8) rmANOVA was conducted with each block as the within-subject variable and group as the between-subject variable; the results that did not meet the assumption of sphericity were adjusted using Greenhouse–Geisser correction. The results showed a significant main effect for block (*F*_(3.7,164)_ = 42.93, *p* < 0.001, *η*^2^ = 0.49), a significant main effect for group (*F*_(1,44)_ = 8.05, *p* = 0.007, *η*^2^ = 0.15), and a nonsignificant interaction between group and block (*F*_(3.7,164)_ = 1.21, *p* = 0.31, *η*^2^ = 0.03). These findings suggest that RGUs performed better overall than participants with IGD in the learning task, but the accuracy of each block did not vary as a function of group. In addition, an independent samples *t*-test was conducted to examine the difference in accuracy between the two groups on the final block. The results showed that although both groups achieved over 90% accuracy, the overall accuracy of the RGUs was significantly higher than that of the IGD group (*t*(45) = −3.08, *p* < 0.001, *η*^2^ = 0.42).

#### Stage 2: Action–outcome test

In the action–outcome test (Fig. 1B), the RGUs (Accuracy = 77%) demonstrated a higher accuracy compared with the IGD group (Accuracy = 69%), but the results of an independent samples *t*-test showed no significant difference between the two groups (*t*(45) = −1.57, *p* = 0.124, *η*^2^ = 0.23), indicating that both groups acquired knowledge of the action and result, and exhibited excellent instrumental learning effects.

#### Stage 3: Slip-of-action test

The final slip-of-action test was used to examine the strategy differences individuals employ when devaluing outcomes (Fig. 1C). With group as a between-subjects variable and outcome type as a within-subjects variable, a 2 (group: IGD, RGU) × 2 (outcome type: valued, devalued) rmANOVA was conducted. The results showed a significant main effect for group (*F*_(1,45)_ = 6.02, *p* = 0.018, *η*^2^ = 0.12), a significant main effect for outcome type (*F*_(1,45)_ = 252, *p* < 0.001, *η*^2^ = 0.85), and a significant interaction effect between group and outcome type (*F*_(1,45)_ = 8.48, *p* = 0.006, *η*^2^ = 0.16). Subsequently, post hoc pairwise comparisons revealed that, for the non-devalued results, the RGU group exhibited significantly higher correct response rates than the IGD group (*F*_(1,45)_ = 9.19, *p* = 0.004, *η*^2^ = 0.17), whereas for the devalued results, the IGD group showed significantly lower correct response rates than the RGU group (*F*_(1,45)_ = 6.22, *p* = 0.016, *η*^2^ = 0.12). Significant differences in the mean response rates between the two groups were found. In further analyses, working memory capacity, correct response rates in the learning phase, action–outcome knowledge in the second learning phase, compulsivity, and impulsivity were used as covariates to explore whether the differences between the two groups were influenced by other factors. When working memory capacity was used as a covariate, significant differences remained in the mean response rates for both non-devalued results (*F*_(1,44)_ = 7.78, *p* = 0.008, *η*^2^ = 0.15) and devalued results (*F*_(1,44)_ = 5.11, *p* = 0.029, *η*^2^ = 0.10) between the two groups. When compulsivity was used as a covariate, significant differences were found in the mean response rates for both non-devalued results (*F*_(1,44)_ = 10.05, *p* = 0.003, *η*^2^ = 0.19) and devalued results (*F*_(1,44)_ = 7.39, *p* = 0.009, *η*^2^ = 0.14) between the two groups. When impulsivity trait was used as a covariate, significant differences were observed in the mean response rates for both non-devalued results (*F*_(1,44)_ = 8.91, *p* = 0.005, *η*^2^ = 0.17) and devalued results (*F*_(1,44)_ = 6.16, *p* = 0.017, *η*^2^ = 0.12) between the two groups. When the level of action–outcome knowledge after the learning phase was used as a covariate, significant differences were found in the correct response rates for non-devalued results (*F*_(1,44)_ = 6.24, *p* = 0.016, *η*^2^ = 0.17), whereas the differences in the mean response rates for devalued results were marginally significant (*F*_(1, 44)_ = 3.8, *p* = 0.058, *η*^2^ = 0.08). These results suggest that action–outcome knowledge has a minimal effect on correct responses for non-devalued results but a greater impact on devalued results.

### EEG results

#### Electrophysiological differences between IGD and RGU

##### Stage 1: Instrumental learning

During the instrumental learning phase, rmANOVA was used to test the differences in the maximum negative wave 220–320 ms after positive and negative feedback in the Fz, Cz, and Pz regions between the IGD and RGU groups. The results revealed significant effects of electrode location (*F*_(1,54)_ = 7.10, *p* = 0.006, *η*^2^ = 0.15) and response type (*F*_(1, 40)_ = 10.35, *p* = 0.003, *η*^2^ = 0.21), whereas the main effect of group was nonsignificant (*F*_(1, 40)_ = 0.01, *p* = 0.921, *η*^2^ < 0.01). No other significant interactions were observed. Given that the differences between electrode locations were not of primary interest, post hoc pairwise comparisons revealed that, at the Fz electrode site, the negative feedback (5.73 ± 7.25 μV) elicited a larger negative wave compared with the positive feedback (9.36 ± 11.81 μV; *t*(41) = −2.16, *p* = 0.037, *η*^2^ = 0.32); at the Cz electrode site, the negative feedback (5.29 ± 7.62 μV) was significantly more negative than the positive feedback (11.03 ± 9.26 μV; *t*(41) = −3.93, *p* < 0.001, *η*^2^ = 0.52); and at the Pz electrode site, the negative feedback (3.83 ± 6.64 μV) produced a larger negative wave compared with the positive feedback (7.70 ± 8.71 μV; *t*(41) = −2.94, *p* = 0.005, *η*^2^ = 0.42).

The results indicate that both groups of participants elicited a larger FRN following negative feedback. However, the intergroup differences were not significant.

##### Stage 3: Slip-of-action test

Stimulus-locked components: First, the analysis of stimulus-locked components is presented. rmANOVA was used to analyze the average amplitude of N2 (see Table 2), including group (IGD, RGU), accompanying responses (inhibition of devalued outcome, response to valuable outcome, slip of action), and electrode locations (Fz, Cz, Pz). The results revealed a significant effect of electrode location (*F*_(1,52)_ = 92.17, *p* < 0.001, *η*^2^ = 0.70), whereas the main effect of the accompanying response was not significant (*F*_(1.5, 61)_ = 2.13, *p* = 0.139, *η*^2^ = 0.05). In addition, the main effect of group (*F*_(1,40)_ = 0.47, *p* = 0.497, *η*^2^ = 0.01) and the interaction effects between the electrode location and group (*F*_(1,52)_ = 0.03, *p* = 0.920, *η*^2^ < 0.01) and between the electrode location and response type (*F*_(1.6,65)_ = 1.76, *p* = 0.185, *η*^2^ = 0.04) were not significant. A significant interaction effect was observed between the accompanying response and group (*F*_(1.5,61)_ = 5.95, *p* = 0.008, *η*^2^ = 0.13). Additionally, a significant three-way interaction among electrode location, accompanying response, and group was found (*F*_(1.6,65)_ = 4.95, *p* = 0.015, *η*^2^ = 0.11). Given that differences in electrode location were not of primary interest, a simple effects analysis was conducted focusing on the interaction effect between the accompanying response and group. Post hoc pairwise comparisons revealed that during the inhibition of devalued outcome, the average waveforms of the IGD group were significantly more negative at the Fz and Cz electrode points (−7.26 ± 6.28 μV; −5.18 ± 5.49 μV) compared with the RGUs (−3.25 ± 4.52 μV; −1.64 ± 4.79 μV), and the differences were statistically significant (*F*_(1,40)_ = 5.54, *p* = 0.024, *η*^2^ = 0.12; *F*_(1,40)_ = 4.92, *p* = 0.032, *η*^2^ = 0.11); there were no statistically significant differences at the Pz electrode point (*F*_(1,40)_ = 2.60, *p* = 0.115, *η*^2^ = 0.06). During the presentation of stimuli accompanying response to the valuable outcome, there were no statistically significant differences at the three electrode points of Fz (*F*_(1,40)_ = 2.13, *p* = 0.152, *η*^2^ = 0.05), Cz (*F*_(1,40)_ = 2.10, *p* = 0.155, *η*^2^ = 0.05), Pz (*F*_(1,40)_ = 0.95, *p* = 0.335, *η*^2^ = 0.02) between the two groups. Similarly, during the presentation of stimuli accompanying slip of action, there were no statistically significant differences at the Fz (*F*_(1,40)_ = 1.61, *p* = 0.212, *η*^2^ = 0.04), Cz (*F*_(1,40)_ = 1.00, *p* = 0.323, *η*^2^ = 0.02), and Pz (*F*_(1,40)_ = 0.03, *p* = 0.865, *η*^2^ < 0.01) electrode points.

**Table 2.** Electrophysiological data and group differences for N200 and P300

		Electrode site	IGD (*n* = 22)	RGU (*n* = 20)	t/χ^2^	*p*
N200	*Response to valuable outcome*	Fz	−5.42 ± 4.85	−3.30 ± 4.53	−1.46	0.152
		Cz	−3.84 ± 4.67	−1.79 ± 4.48	−1.45	0.155
		Pz	1.57 ± 4.44	1.72 ± 3.34	−0.98	0.335
	*Inhibitions to devalued outcome*	Fz	−7.26 ± 6.29	−3.25 ± 4.52	−2.35	0.024
		Cz	−5.18 ± 5.48	−1.64 ± 4.79	−2.22	0.032
		Pz	0.22 ± 4.50	2.29 ± 3.73	−1.61	0.115
	*Slip of action*	Fz	−4.96 ± 7.69	−8.59 ± 10.72	1.27	0.212
		Cz	−3.33 ± 6.71	−5.74 ± 8.81	1.00	0.323
		Pz	0.82 ± 6.56	0.52 ± 4.53	0.17	0.865
P300	*Response to valuable outcome*	Fz	−8.06 ± 4.40	−6.34 ± 6.43	−1.02	0.316
		Cz	−5.67 ± 4.44	−4.09 ± 6.27	−0.95	0.347
		Pz	0.75 ± 4.44	1.59 ± 4.40	−0.62	0.539
	*Inhibitions to devalued outcome*	Fz	−7.11 ± 5.70	−4.47 ± 6.30	−1.42	0.162
		Cz	−4.09 ± 5.01	−1.71 ± 6.72	−1.31	0.199
		Pz	2.64 ± 4.69	3.61 ± 5.37	−0.63	0.536
	*Slip of action*	Fz	−8.11 ± 6.30	−8.76 ± 10.61	0.25	0.806
		Cz	−4.91 ± 5.55	−6.68 ± 9.23	0.76	0.451
		Pz	1.86 ± 4.88	−0.17 ± 4.88	1.35	0.186

IGD: Internet gaming disorder; RGU: Recreational game user.

**Fig. 2.**
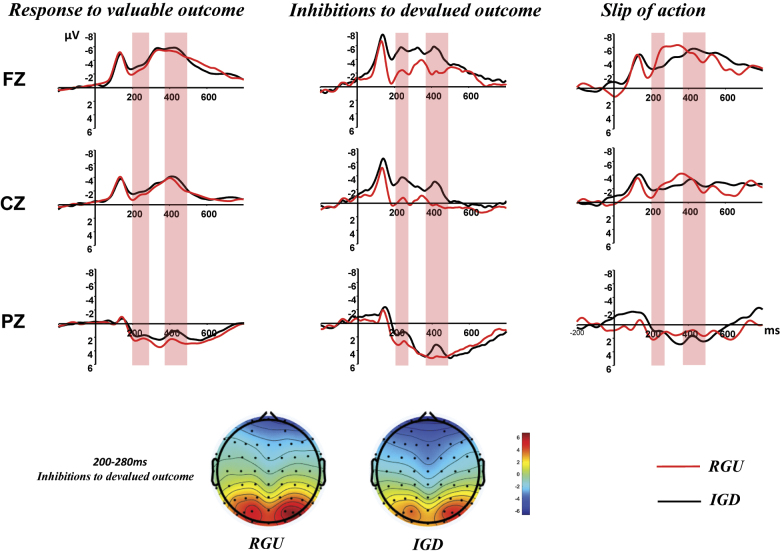
Stimulus-locked ERPs during the slips-of-action test between IGD (black line) and RGU (red line). ERPs at Fz, Cz and Pz time-locked to stimuli preceding responses to valuable outcomes preceding slips of action and preceding inhibitions to devalued outcomes. Topographies depict the differences between stimuli preceding inhibitions to devalued at the indicated time windows

Additionally, an rmANOVA was employed to analyze the average amplitude of P3 (see Table 2). The results revealed a significant effect of electrode location (*F*_(1,50)_ = 119.88, *p* < 0.001, *η*^2^ = 0.75). The main effect of accompanying response was significant (*F*_(1.7,67)_ = 6.63, *p* = 0.004, *η*^2^ = 0.14), whereas the main effect of group was not significant (*F*_(1,40)_ = 0.19, *p* = 0.669, *η*^2^ < 0.01). The interaction effect between the electrode location and group was nonsignificant (*F*_(1,50)_ = 0.62, *p* = 0.467, *η*^2^ = 0.02), as was the interaction effect between the electrode location and response type (*F*_(2,81)_ = 0.68, *p* = 0.511, *η*^2^ = 0.02). The interaction effect between the accompanying response and group approached significance (*F*_(1.7,67)_ = 3.22, *p* = 0.055, *η*^2^ = 0.08), whereas the three-way interaction among electrode location, accompanying response, and group was not significant (*F*_(2,81)_ = 0.32, *p* = 0.727, *η*^2^ < 0.01). Given that differences in electrode location were not the focus of interest, a simple effects analysis was conducted on the interaction between the accompanying response and group. Post hoc pairwise comparisons revealed that there were no statistically significant differences between the two groups in the three accompanying responses: inhibition of devalued outcome: Fz (*F*_(1,40)_ = 2.03, *p* = 0.162, *η*^2^ = 0.05), Cz (*F*_(1,40)_ = 1.71, *p* = 0.199, *η*^2^ = 0.04), Pz (*F*_(1,40)_ = 0.39, *p* = 0.536, *η*^2^ = 0.01); response to valuable outcome: Fz (*F*_(1,40)_ = 1.03, *p* = 0.316, *η*^2^ = 0.03), Cz (*F*_(1,40)_ = 0.91, *p* = 0.347, *η*^2^ = 0.02), Pz (*F*_(1,40)_ = 0.38, *p* = 0.539, *η*^2^ = 0.01); slip of action: Fz (*F*_(1,40)_ = 0.06, *p* = 0.806, *η*^2^ < 0.01), Cz (*F*_(1,40)_ = 0.58, *p* = 0.451, *η*^2^ = 0.01), Pz (*F*_(1,40)_ = 1.81, *p* = 0.186, *η*^2^ = 0.04) (Fig. 2).

Response-locked components: We analyzed the response-locked components as follows. We compared the ERN and P_E_ components after habitual responses (continuation of responding to devalued outcome) between the IGD and RGU groups (see Table 3). Initially, we compared the differences in average amplitudes of 20–80 ms and 350–500 ms following action-slip responses for the two groups. For the ERN component, independent samples *t*-tests indicated no significant differences between the two groups: Fz (*t*(40) = 1.38, *p* = 0.176, *η*^2^ = 0.21), Cz (*t*(40) = 1.10, *p* = 0.280, *η*^2^ = 0.17), Pz (*t*(40) = 1.12, *p* = 0.269, *η*^2^ = 0.17). Similarly, no significant differences were found for the P_E_ component: Fz (*t*(40) = 0.82, *p* = 0.415, *η*^2^ = 0.13), Cz (*t*(40) = 1.11, *p* = 0.276, *η*^2^ = 0.17), Pz (*t*(40) = 1.08, *p* = 0.288, *η*^2^ = 0.17). Subsequently, we compared the maximum negative peaks in 20–80 ms and the maximum positive peaks in 350–500 ms for the two groups. The results indicated no significant differences in peak values for the ERN component: Fz (*t*(40) = 1.55, *p* = 0.129, *η*^2^ = 0.24), Cz (*t*(40) = 1.53, *p* = 0.132, *η*^2^ = 0.24), Pz (*t*(40) = 1.63, *p* = 0.111, *η*^2^ = 0.25). Similarly, no significant differences were observed for the peak values of the PE component: Fz (*t*(40) = 0.33, *p* = 0.741, *η*^2^ = 0.05), Cz (*t*(40) = 0.67, *p* = 0.502, *η*^2^ = 0.11), Pz (*t*(40) = 0.53, *p* = 0.598, *η*^2^ = 0.08).

**Table 3.** Group differences in mean and peak amplitude for ERN and P_E_

		Electrode site	IGD (*n* = 22)	RGU (*n* = 20)	t/χ^2^	*p*
ERN	Mean amplitude	Fz	4.27 ± 6.93	1.56 ± 5.69	1.38	0.176
		Cz	2.05 ± 6.42	0.03 ± 5.45	1.10	0.280
		Pz	−1.90 ± 5.25	−3.79 ± 5.66	1.12	0.269
	Peak amplitude	Fz	1.22 ± 6.62	−2.09 ± 7.22	1.55	0.129
		Cz	−0.06 ± 6.55	−3.19 ± 6.67	1.54	0.132
		Pz	−4.01 ± 5.43	−7.06 ± 6.70	0.63	0.111
P_E_	Mean amplitude	Fz	8.22 ± 6.35	6.17 ± 9.63	0.82	0.415
		Cz	8.01 ± 6.31	4.80 ± 11.89	1.11	0.276
		Pz	2.72 ± 6.88	−0.24 ± 10.69	1.08	0.288
	Peak amplitude	Fz	13.64 ± 7.94	12.72 ± 10.03	0.33	0.741
		Cz	12.41 ± 7.33	10.30 ± 12.40	0.68	0.502
		Pz	6.61 ± 7.47	5.09 ± 10.93	0.53	0.598

IGD: Internet gaming disorder; RGU: Recreational game user.

In summary, the results suggest that following stimulus presentation, when the accompanying response involves the suppression of devalued outcomes, the IGD group exhibited greater activation at the Fz and Cz electrode sites compared with the RGU group, and this difference was statistically significant. By contrast, no significant differences were observed between the two groups at any electrode sites when the accompanying response involved reactions to valued outcomes and action slips. There was no significant difference in the response-locked components ERN and P_E_ between the two groups after the occurrence of a slip of action.

#### Electrophysiology of goal–directed versus habitual system

**Fig. 3.**
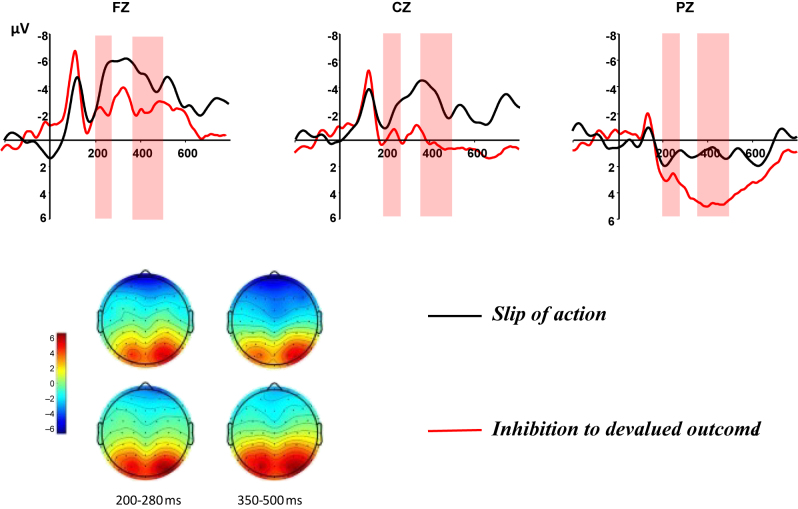
Stimulus-locked ERPs during the slips-of-action test. ERPs at Fz, Cz and Pz time-locked to stimuli preceding slips of action (black line) and preceding inhibitions to devalued outcomes (red line). Topographies depict the differences between stimuli preceding inhibitions to devalued and slips of action at the indicated time windows

**Fig. 4.**
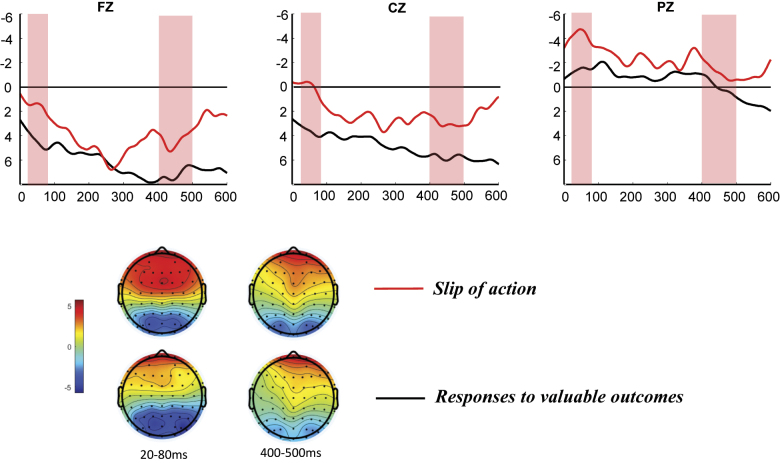
Response-locked ERPs during the slips-of-action test. ERPs at Fz, Cz and Pz time-locked to responses to valuable outcomes (black line) and slips of action (red line). Topographies depict the differences between slips of action and responses to valuable outcomes at the indicated time windows

**Fig. 5.**
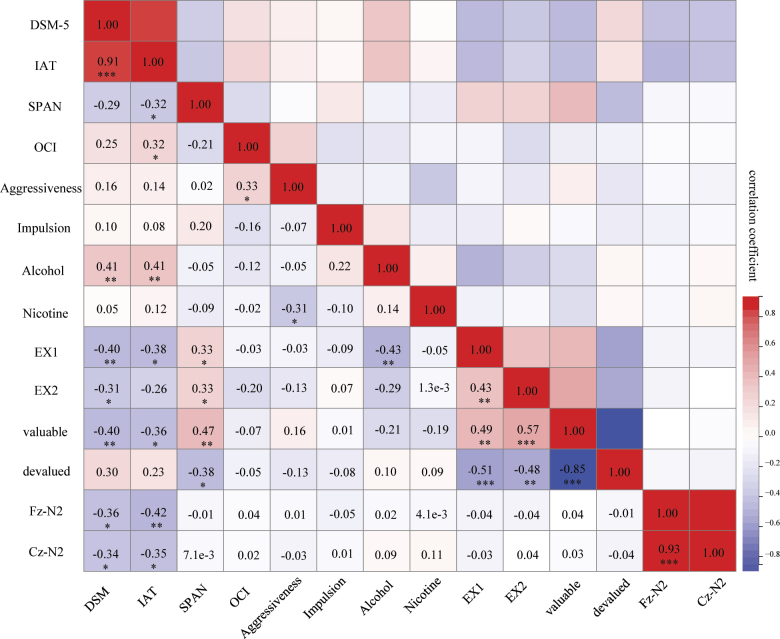
The correlation between various variables. DSM-5: IGD Diagnostic scale from Diagnostic and Statistical manual of Mental Disorders-5; IAT: Internet addiction test; SPAN: Working memory span; OCI: Degree of obsessive-compulsive disorder; EX1: Correct response of the last block in instrumental learning phase; EX2:Correct response during the action-outcome test; Fz-N2: The N2 mean amplitude at the Fz accompanied by the inhibition to the devalued outcome; Cz-N2: The N2 mean amplitude at the Cz accompanied by the inhibition to the devalued outcome. *p < 0.05, **p < 0.01, ***p < 0.001

To investigate the differences between habitual control and goal-oriented behavior, we exclusively focused on RGU players at this stage. This analyze examined the differences between stimulus-locked and response-locked measures in inhibitory success and slip of action following the presentation of devalued outcome-related stimuli (Figs 3–5).

##### Stimulus-locked components

In this phase, we conducted an rmANOVA to assess the effects of devaluation-related stimuli on subsequent responses (inhibition of devaluation outcomes, slip of action) and electrode locations (Fz, Cz, Pz), to explore the differences in N2 and P3 component amplitudes associated with habitual control and goal–directed behavior. The rmANOVA results for the N2 component revealed significant main effects of subsequent responses (*F*_(1,19)_ = 5.08, *p* = 0.036, *η*^2^ = 0.21) and electrode location (*F*_(1,24)_ = 34.67, *p* < 0.001, *η*^2^ = 0.65), as well as a marginally significant interaction between electrode location and subsequent responses (*F*_(1,21)_ = 4.07, *p* = 0.052, *η*^2^ = 0.21). Post hoc pairwise comparisons indicated that, after controlling for electrode location, a significant difference was observed at Fz, where the N2 amplitude elicited by action slips (−8.59 ± 10.72 μV) was significantly more negative compared with the inhibition of devaluation outcomes (−3.25 ± 4.52 μV; *F*_(1,19)_ = 5.22, *p* = 0.034, *η*^2^ = 0.22). Similarly, at Cz, the N2 amplitude elicited by action slips (−5.74 ± 8.81 μV) was significantly more negative compared with the inhibition of devaluation outcomes (−1.64 ± 4.79 μV; *F*_(1,19)_ = 5.04, *p* = 0.037, *η*^2^ = 0.21). However, this difference was not observed at Pz (*F*_(1,19)_ = 2.94, *p* = 0.103, *η*^2^ = 0.13).

The rmANOVA for the P3 component revealed significant effects of subsequent responses (*F*_(1,19)_ = 7.44, *p* = 0.013, *η*^2^ = 0.28) and electrode location (*F*_(1,24)_ = 32.66, *p* < 0.001, *η*^2^ = 0.63), with no significant interaction between electrode location and subsequent responses (*F*_(1,20)_ = 0.43, *p* = 0.536, *η*^2^ = 0.02). Post hoc pairwise comparisons indicated that, after controlling for electrode location, there was no significant difference in P3 amplitude between inhibition of devaluation outcomes and action slips at Fz (*F*_(1,19)_ = 3.84, *p* = 0.065, *η*^2^ = 0.17). However, at Cz and Pz, the P3 amplitude elicited by action slips (−6.68 ± 9.23 μV; −0.17 ± 4.87 μV) was significantly lower compared with the inhibition of devaluation outcomes (−1.71 ± 6.72 μV; 3.61 ± 5.37 μV; *F*_(1,19)_ = 7.94, *p* = 0.011, *η*^2^ = 0.30; *F*_(1,19)_ = 10.35, *p* = 0.005, *η*^2^ = 0.35).

The results indicate that when subsequent responses are habitual, larger N2 and P3 amplitudes are observed compared with goal–directed responses.

##### Response-locked components

In this stage, we further examined the feedback-locked ERN and P_E_ components following habitual responses (continuing responses to devaluation results), comparing the maximum negative peaks, average wave amplitudes between 20 and 80 ms, as well as the maximum positive peaks and average wave amplitudes between 350 and 500 ms of responses to valuable outcomes and slips of action. The paired-samples *t*-test revealed significant differences in the average ERN amplitudes between the two types of responses, with significant differences observed at the Fz and Cz electrode sites: Fz (*t*(19) = 2.16, *p* = 0.044, *η*^2^ = 0.44), Cz (*t*(19) = 2.21, *p* = 0.04, *η*^2^ = 0.45), and Pz (*t*(19) = 1.93, *p* = 0.068, *η*^2^ = 0.40).

Analysis of peak amplitudes revealed that habitual responses elicited larger negative peaks in the ERN component compared with the correct responses to non-devalued outcomes, with significant differences observed at the Fz, Cz, and Pz (Fz: *t*(19) = 2.86, *p* = 0.01, *η*^2^ = 0.55; Cz: *t*(19) = 3.06, *p* = 0.006, *η*^2^ = 0.58; Pz: *t*(19) = 3.53, *p* = 0.002, *η*^2^ = 0.63). No significant differences were observed for either the average amplitude or the maximal positive peak in the P_E_ component.

The results indicate that following action-slip responses, a more negative ERN is observed compared with the correct responses to devalued outcomes, whereas no significant differences between the two types of responses were observed in the P_E_ component.

### Relationship between behavioral and EEG data

Pearson correlation was employed to examine the relationships between variables. The results revealed a significant positive correlation between DSM-5 and IAT scores (*r* = 0.91, *p* < 0.01), indicating the validity of the measurement tools. Concerning behavioral performance, the scores obtained in the IAT exhibited statistically significant negative correlations with task performance during the instrumental contingency reflex learning phase (*r* = −0.4, *p* < 0.01), along with significantly negative correlations with accurate responses to non-devalued outcomes in the slip-of-action test (*r* = −0.39, *p* < 0.01). Regarding electrophysiological performance, the scores derived from the IAT displayed significant negative correlations with the average N2 wave amplitude observed during the inhibition of devalued outcomes in the slip-of-action test, as evidenced by the readings at the Fz (*r* = −0.39, *p* < 0.01) and Cz (*r* = −0.31, *p* < 0.05). Overall, the findings suggest that the degree of IGD appears to impede individual performance in reinforcement learning tasks, consequently compromising the integrity of the goal–directed system.

## Discussion

### Impaired learning ability in participants with IGD

Time-locked event-related potentials are a useful tool for studying real-time brain activity (Park, Kim, Kim, & Choi, 2017). To our knowledge, this study provides the first electrophysiological evidence for how the dual systems of habitual control and goal–directed behavior are characterized in individuals with IGD and RGUs. We used an outcome devaluation paradigm to test the differences between the two groups, where participants needed to first establish a habit (corresponding to a reinforcement learning phase), and then break the established habit (corresponding to a slip-of-action test). The behavioral results are similar to previous studies on substance addiction and behavioral addiction (Ersche et al., 2016; Sjoerds et al., 2013; Zhou et al., 2018). During the process of establishing habitual responses, we provided monetary points as rewards, and the results showed that although the learning performance of both groups steadily improved and remained at a high level, the performance of habitual response establishment in the IGD group was weaker than that in the RGU group, indicating that excessive indulgence in online games may affect an individual's reinforcement learning ability related to rewards. Typically, substance addiction (Ekhtiari, Victor, & Paulus, 2017; Verdejo-García, Lawrence, & Clark, 2008) and behavioral addiction (Clark, Boileau, & Zack, 2019; Lei et al., 2022) are associated with a weakening of reinforcement learning related to rewards. This could be more specific to reflect the tendency for weakened reinforcement learning specific to rewards beyond the addictive behavior (in this case, gaming). Albeit seemingly minor, this is an important distinction given current shortfalls (both theoretical and methodological) in properly distinguishing between addiction-related vs natural rewards in the broader literature concerning behavioral addictions.

At the electrophysiological level, data from the learning phase confirmed that both the IGD and RGU groups exhibited a significant negative deflection component, the FRN, following negative feedback. This finding is consistent with previous studies, which indicate that FRN elicited by feedback related to negative outcomes is significantly more negative compared with feedback associated with positive rewards (Holroyd & Coles, 2002; O'Doherty, Dayan, Friston, Critchley, & Dolan, 2003; Yu & Zhou, 2006). However, our study found no significant differences in FRN between the IGD and RGU groups. In addiction research, individuals with addiction often exhibit abnormal increases or decreases in FRN following both positive and negative feedback related to natural rewards. Such abnormal patterns of FRN reflect dysfunctions in the reward system (Walsh & Anderson, 2012). The lack of observed group differences may be explained by the fact that previous studies have employed tasks such as simple gambling tasks, reward expectancy violation tasks, balloon analog risk tasks, and probability reversal learning tasks (He et al., 2017; Hixson, Burkhouse, Gorka, & Klumpp, 2019; Torres et al., 2013; Yau et al., 2015), which focus on reward functions in addiction. However, our devaluation task emphasized the learning process and the matching of stimuli–response–outcome, with feedback aimed at enhancing learning rather than the reward. Consequently, the characteristics of our task may have led to the lack of significant differences in FRN between the two groups.

### Differences between the IGD and RGU groups

The results of the slip-of-action test revealed that IGD participants, compared with RGUs, showed more responses to devalued outcomes, which is a core feature of habitual control: insensitivity to outcome devaluation (Wood & Ruenger, 2016). Individuals with IGD are more inclined to rely on automated habitual responses rather than deliberate decision-making processes when facing decisions (Dong et al., 2021; Zhou et al., 2021).

The electrophysiological results explain the reasons for this tendency. This study found that the difference between IGD and RGU groups manifested in the successful inhibition stage, where IGD activated a larger N2. The N2 component is a negative potential, often considered to be related to inhibitory control ability. This finding suggests that compared with RGU, individuals with IGD require more cognitive resources and effort to achieve correct results in inhibitory control tasks, which may be closely related to their decreased inhibitory control ability (Li et al., 2017). The results of correlation analyses support this conclusion, indicating that the severity of IGD can predict the average amplitude of N2 and P3 during the correct inhibition phase, suggesting that more severe IGD reduces individual ability to activate goal–directed behavior.

Current research in the field has shown that individuals with IGD exhibit higher error rates and longer response times in inhibitory control tasks (Dong, Wang, Du, & Potenza, 2018; Duven, Müller, Beutel, & Wölfling, 2015; Littel et al., 2012). This may further suggest that their ability to maintain self-control in the face of external interference is impaired. In neuroimaging studies, functional connectivity of brain areas related to inhibitory control, such as the dorsolateral prefrontal cortex and anterior insula, show impairment in individuals with IGD (Gentile et al., 2011; Meng, Deng, Wang, Guo, & Li, 2015); this may lead to difficulties in executing inhibitory tasks for these individuals.

Inconsistent with our hypothesis, no significant differences were observed between the two groups regarding the P3 component. This result contrasts sharply with other studies that have reported differences in P3 amplitude in similar go/no-go tasks (Dong, Lu, Zhou, & Zhao, 2010; Zhou, Yuan, Yao, Li, & Cheng, 2010). However, some studies have found that there is no significant difference in P3 amplitudes between excessive computer gamers and control groups using the go/no-go paradigm (Littel et al., 2012). These results suggest that IGD participants may not experience any difficulties in inhibition at later stages.

Furthermore, no significant differences were found between IGD and RGU groups in the slip-of-action test, whether in the presentation of stimuli or after the response, which is inconsistent with our hypothesis. This suggests that the imbalance in the dual systems of IGD participants may occur during the early stages of habit formation (Lally & Gardner, 2013) and the breaking of established habits (Duhigg & Organizations, 2013), whereas once habits are established, individuals with IGD may function similarly to the average person.

In conclusion, the differences between the IGD and RGU groups are evident in goal–directed behavior, with a larger N2 recorded in individuals with IGD compared with RGUs, indicating greater attentional resources being used. This suggests that the inhibitory control and cognitive flexibility of IGD individuals are impaired, leading to greater difficulty in initiating goal–directed behavior (Wood & Ruenger, 2016), as it requires more cognitive resources for thoughtful consideration (Dong et al., 2021; Zhou et al., 2021). These findings provide clues for further research on the neural mechanisms and treatment methods of IGD.

### Electrophysiology of goal–directed versus habitual system

The investigation targeting RGU yielded several intriguing findings. We compared the N2 and P3 components of two types of RGU responses under different stimulus presentations. In contrast to a previous study (Yousuf et al., 2019), we found that the N2 amplitude associated with slips of action was significantly larger than that of successfully inhibited responses. In other words, compared with goal–directed behavior, stimuli related to habit seemed to elicit a larger N2 amplitude.

Habitual control is often associated with automation and unconsciousness (Mazar & Wood, 2018), whereas goal–directed behavior reflects the ability to adapt to a constantly changing environment (Huster et al., 2013). In go/no-go tasks, no-go trials activate a larger N2 amplitude compared with go trials due to increasing conflict responses (Botvinick et al., 2001; Donkers & van Boxtel, 2004; Smith & Randall, 2012). The larger amplitude of habitual responses based on the slip-of-action test reflects the increased conflict in this process. Previous research has revealed that compared with successful inhibition, failed inhibition during the execution control process is associated with larger N2 amplitudes (Bartholow et al., 2005; Huster et al., 2013; Kamarajan et al., 2009). In other words, the brain requires more resources to process conflict monitoring and correct errors or adjust behavior during failed inhibition. However, some research has shown that successful inhibition activates larger N2 amplitudes (Luus et al., 2007; Roche, Garavan, Foxe, & O'Mara, 2005; Schmajuk, Liotti, Busse, & Woldorff, 2006).

Similarly, the increase in the average P3 amplitude is related to habit. The P3 component usually reflects psychological processes such as attention, expectation, attribution, and processing of new and different stimuli (Picton, 1992; Pritchard, 1981). In go/no-go tasks, failed inhibition induces a larger average P3 amplitude compared with successful inhibition (Kok et al., 2004; Roche et al., 2005). In our experiment, the change in P3 amplitude reflects that additional cognitive resources are required to process errors or somewhat unexpected events.

Then, we compared the ERN and P_E_ components of habitual responses in the response-locked phase. The results were consistent with our expectations, with a larger negative wave observed after habitual responses (Gehring & Fencsik, 2001; Larson, Clayson, & Baldwin, 2012). ERN is an electroencephalogram component closely related to error responses and executive control (Kirschner & Klein, 2022; Nunez-Estupinan et al., 2022). Habitual responses accompanied by a slip of action are essentially erroneous behavioral responses, and effective error monitoring and adaptation after errors are crucial for adjusting future behavior (Ullsperger, Fischer, Nigbur, & Endrass, 2014). Therefore, even if individual error monitoring mechanisms are activated after habitual responses, habitual deviations remain inevitable. In our experiment, habit seems to be related to the failure of behavioral inhibition and the intensification of conflicts in consciousness, which means doing something despite knowing it is not right, leading to the occurrence of habitual responses. No significant differences were observed in the P_E_ component; similarly, previous studies did not find notable variations in the P_E_ component (Yousuf et al., 2019). The P_E_ component reflects the conscious processing of errors (Shalgi, Barkan, & Deouell, 2009), suggesting that error monitoring associated with habitual responses may occur at earlier stages.

Overall, contrary to our hypothesis, we found that habitual control seems to be associated with larger N2 and P3 amplitudes in the stimulus-locked phase. Compared with goal–directed behavior, habit is accompanied by more conscious activity and cognitive resource consumption; therefore, habit may not be an unconscious behavior (Wood & Ruenger, 2016), but an automated behavior intensified by conflicts in consciousness. In the response-locked phase, we found that habitual responses induce a larger ERN, which partly confirms the above findings.

## Limitations and future directions

### Limitations

This study employed the event-related potential technique to focus on the electrophysiological components associated with habitual and goal–directed behaviors. Additionally, it compared the electrophysiological differences between IGD and RGU participants within the dual–systems framework. Although our study provides preliminary insights into the dual–system electrophysiological components and the inhibitory function abnormalities in IGD, several limitations need to be addressed. First, the sample in this study consisted of university students, which limits the generalizability and reliability of the findings. Future research should expand the sample to include a more diverse population so that the robustness of the conclusions is enhanced. Second, although the current study primarily employed outcome devaluation tasks, habits and goal–directed behaviors established in the laboratory may differ in mechanisms and manifestations from those in daily life. Future research should employ ecological methods to reveal the real–time brain activity of IGD individuals during habitual and goal–directed behaviors. Additionally, the study considered only a subset of covariates. In the context of addictive behaviors, dimensions related to personal traits (e.g., trait compulsivity and lack of perseverance) may be particularly relevant to habitual and goal–directed behaviors. Beyond that, other scales for the measured covariates may have yielded different results. Although this is always true for measurement selection, it is particularly relevant here given that compulsivity was measured with the OCI (which specifically measures OCD-related symptoms, rather than measuring trait compulsivity as a transdiagnostic dimension (Hook et al., 2021). Finally, IGD is a complex phenomenon involving multiple cognitive and emotional processes. Our study focused solely on habitual control and goal–directed behavior. Future research should employ a variety of tasks and experimental paradigms to gain a comprehensive understanding of the electrophysiological characteristics of individuals with IGD.

### Future directions

First, the outcome devaluation paradigm can incorporate game-specific cues. Considering the heightened cue reactivity observed in individuals with IGD, this approach may produce intriguing outcomes. Second, the research sample should be broadened to include cross-cultural and diverse populations. This expansion would facilitate a deeper understanding of the electrophysiological characteristics of individuals with IGD across various populations and their potential associations with cultural and social factors. Additionally, incorporating other neuroimaging techniques, such as fMRI, functional near–infrared spectroscopy and magnetoencephalography, is essential for a comprehensive understanding of the brain activity characteristics and underlying mechanisms of IGD individuals. Finally, employing longitudinal designs and long–term tracking methods would enable the exploration of the developmental trajectory and enduring effects of IGD.

## Conclusion

In this study, we compared electrophysiological differences across three response types between individuals with IGD and RGUs. The results indicated that individuals with IGD demonstrated significantly greater activation during successful inhibition responses, suggesting that they may face greater challenges in inhibitory control. This implies that individuals with IGD are more prone to adopting habitual behaviors and exhibit diminished goal–directed actions. Consequently, the inhibitory control and cognitive flexibility of individuals with IGD may be compromised, requiring increased willpower for deliberate thought and resulting in a greater tendency toward habitual behaviors. Simultaneously, we found that habitual behaviors were associated with heightened N2 and P3 amplitudes, whereas goal–directed behaviors corresponded with reduced N2 and P3 amplitudes, contrary to earlier studies. In our study, habitual behaviors might have diverged from the previously recognized unconscious, automatic behaviors, indicating that they may consume more cognitive resources and necessitate additional attention to address conflicts between subjective intentions and actual actions. Our research aims to enhance the understanding of the neural mechanisms underlying IGD. Future prevention and intervention strategies for IGD could focus on strengthening individuals' goal–directed systems, such as by placing goals in prominent locations or using reminders, to attenuate the effects of habitual behaviors. Concurrently, habit–based interventions, including cue exposure therapy, may emerge as effective strategies for addressing IGD in future treatments.

## Supplementary material
