# Supplementary material for: Increasingly dependent on habit? A study on the electrophysiological mechanisms of goal–directed and habitual control in internet gaming disorder
Source: J Behav Addict. 2025 May 15;14(2):757–75. doi: 10.1556/2006.2024.00084 (PMC12231473; doi:10.1556/2006.2024.00084)
Supplement: Supplementary file 1 [file jba-14-757-s001.pdf]

Gao, X. et al.: Increasingly Dependent on Habit? A Study on the Electrophysiological Mechanisms of Goal–directed and Habitual Control in Internet Gaming Disorder.

https://doi.org/10.1556/2006.2024.00084

Supplementary material

				Response to valuable outcome_stimula						Inhibition of devalued outcome_stimula						Slip of action_stimula						Slip of action_response											
				N200			P300			N200			P300			N200			P300			ERN-average			ERN-peak			PE-average			PE-peak		
Subject	Team	Accepted (%)	Interpolate channels	Fz	Cz	Pz	Fz	Cz	Pz	Fz	Cz	Pz	Fz	Cz	Pz	Fz	Cz	Pz	Fz	Cz	Fz	Fz	Cz	Pz	Fz	Cz	Pz	Fz	Cz	Pz	Fz	Cz	Pz
1	IGD	91.70		1.67	2.39	4.39	-2.98	-2.36	1.57	-3.58	-2.38	-0.29	-5.47	-3.59	0.17	-3.93	1.39	4.39	-8.49	-1.95	4.09	-12.35	-16.01	-17.44	-15.53	-19.64	-19.41	3.8	5.23	2.97	11.71	10.84	7.76
2	IGD	100.00		-10.45	-10.63	6.26	-11.25	-5.47	9.26	-9.53	-7.7	8.98	-9.77	-2.11	13.4	0.67	-1.38	6.26	-7.52	-0.43	11.54	-1.82	-6.18	-9.05	-4.75	-10.43	-14.66	12.47	9.18	-0.15	22.87	16.82	5.84
3	IGD	93.00		-9.32	-10.52	-3.79	-6.46	-6.28	0.76	0.21	-1.14	3.49	4.13	3.29	5.95	-6.84	-2.7	3.62	-1.04	-0.94	2.86	2.99	-5.22	-8.32	-8.03	-6.31	-9.82	-2.74	5.29	-4.12	4.15	11.77	1.53
4	IGD	100.00		3.27	6.47	9.64	-0.57	3.9	8.49	-0.37	2.3	5.65	-4.81	0.53	5.77	2.31	2.19	9.03	-0.17	0.49	9.48	8.12	5.03	1.86	5.83	3.61	0.91	4.32	2.34	-2.33	6.87	4.29	-0.4
5	IGD	91.20		-13.73	-10.08	-1.75	-15.98	-12.29	-1.59	-18.88	-15.91	-4.81	-14.16	-12.8	-2.75	-23.42	-21.93	-10.37	-20.16	-16.39	-1.8	12.44	6.02	-4.98	10.35	4.02	-6.95	17.05	16.21	7.74	25.83	21.67	11.87
6	IGD	99.50		-4.62	-1.65	1.08	-5.75	-0.24	6.64	-6.02	-3.79	-1.91	-1.52	3.16	8.78	-10.52	-5.8	-0.55	-8.53	-4.98	3.08	0.21	0.47	-4.9	-1.26	-0.98	-9	8.29	8.51	5.31	13.27	12.91	7.44
7	IGD	100.00		-3.9	-2.33	0.37	-7.98	-6.27	-0.1	-7.67	-4.82	-1.78	-9.24	-4.74	1.3	-3.9	-0.7	2.46	-4.4	-1.93	3.45	11.27	6.36	2.29	7.03	4.65	0.69	7.81	5.87	3.2	18.16	10.43	6.27
8	IGD	99.50		-10.57	-5.44	-1.04	-9.15	-1.99	3.61	-13.65	-8.7	-2.95	-12.73	-5.56	2.44	-10.29	-4.59	1.53	-6.56	0.69	6.76	7.34	4.8	3.85	2.35	1.31	1.98	11.71	10.51	8.62	14.48	13.4	12.73
9	IGD	89.80		-13.67	-11.63	-6.56	-11.61	-10.14	-5.46	-16.07	-13.81	-8.31	-11.28	-8.01	-1.29	-20.58	-17.87	-19.28	-19.98	-13.09	-4.78	15.65	15.29	7.03	8.47	9.84	-0.18	21.52	24.99	20.1	32.72	33.18	28.11
10	IGD	85.30		-7.33	-4.33	-0.11	-12.02	-9.23	-4.09	-2.43	0.15	4.28	-5.12	-0.87	5.01	7.54	6.68	9.81	-1.08	0.62	6.92	-3.39	-2.48	-1.99	-4.92	-4.04	-3.3	-0.85	-1.49	-3.55	1.9	2.25	-1.75
11	IGD	62.50	M1.M2																														
12	IGD	100.00		-2.83	-4.15	-3.84	-6.19	-4.99	-1.96	-9.05	-8.08	-3.61	-10.13	-6.67	0.42	-8.94	-7.31	1.37	-10.03	-6.91	0.81	4.25	1.59	-2.16	2.06	-0.76	-3.83	8.4	7.44	2.47	10.72	9	4.21
13	IGD	97.80		-8.45	-6.3	-0.54	-8.28	-5.07	2.9	-13.93	-11.91	-3.54	-13.81	-11.01	-0.03	-11.01	-8.94	-3.84	-11.47	-8.62	-0.1	5.17	4.23	-1.06	4.43	3.52	-1.67	7.36	7.1	2.13	9.12	9.07	3.84
14	IGD	100.00		-0.17	0.39	3.76	-0.39	0.08	4.04	2.91	2.62	6.08	6.12	4.64	7.09	4.26	2.7	6.98	4.41	3.71	7.22	2.66	3.48	1.1	0.99	2	0.96	4.83	4.09	-1.81	10.09	9.06	3.4
15	IGD	98.40		-2.05	-0.23	1.11	-7.24	-5.57	-3.58	-0.29	0.81	1.78	-3.41	-3.78	-2.08	-5.3	-3.11	-0.98	-11.8	-8.35	-5.1	4.26	3.94	2.16	3.09	2.75	1.15	10.85	10.09	9.12	16.54	15.59	11.06
16	IGD	100.00		-3.97	-2.38	2.89	-11.08	-10.42	-1.43	-6.35	-2.9	3.06	-14.07	-11.05	-2.81	-3.93	-4.32	0.2	-9.48	-10.56	-2.51	6.72	7.72	-1.34	5.62	6.74	-2.86	6.62	7.53	-2.26	9.94	11.35	2.06
17	IGD	100.00		-0.65	0.33	1.11	-1.87	-2.07	1.47	-2.17	-2.27	-0.85	-1.66	-0.94	3.01	-4.4	-2.56	-1.66	-6.95	-3.87	0.06	-3.56	-2.52	-5.49	-4.86	-4.06	-6.5	4.81	5.83	1.88	7.37	10.52	6.37
18	IGD	92.10		-5.32	-2.43	3.1	-13.04	-12.27	-5.71	-10.95	-4.88	4.73	-15.05	-12.04	-3.07	-5.99	-2.13	5.42	-18.89	-13.38	-3.29	10.38	8.19	2.11	5.64	3.97	-1.93	5.69	-0.74	-8.43	11.64	4.37	-2.84
19	IGD	95.00	T7.T8	0.44	0.81	3.01	-7.78	-6.42	-0.97	-10.34	-5.84	3.03	-7.09	-1.8	8.51	-7.38	0.25	5.82	-10.61	-0.38	7.04	6.69	3.9	-1.01	7.05	5.2	-3.86	8.57	9.82	7.12	10.49	11.65	9.6
20	IGD	95.80		-10.62	-5.09	3.68	-7.4	-2.79	5.7	-14.78	-11.97	-5.27	-6.86	-6.3	0.98	-6.84	-6.94	-4.63	-9.3	-9.05	-4.88	-7.06	-4.8	-3.01	-9.02	-6.51	-3.13	-1	-0.38	-10.16	5.86	3.63	-6.3
21	IGD	71.50	M1.M2																														
22	IGD	97.90		-8.58	-8.9	-3.88	-13.12	-12.94	-5.62	-11.11	-9.49	-1.47	-8.73	-2.97	8.47	1.5	-3.85	-1.63	-10.2	-10.31	-2.84	2.13	3.05	2.71	-0.16	1.36	1.52	9.04	8.56	6.37	11.18	10.61	8.16
23	IGD	99.50		-5.61	-5.84	0.49	-13.67	-8.8	3.73	-8.67	-7.88	-5.03	-8.36	-7.21	-3.86	4.68	4.96	-0.89	-3.79	-0.97	-0.31	13.57	2.17	-3.37	6.56	-2.19	-5.42	10.09	9.88	3.14	16.28	13.37	7.02
24	IGD	98.60		-2.77	-2.96	-0.86	-3.43	-3.2	-1.19	3.05	3.62	3.55	-3.35	-0.09	2.57	3.24	2.59	4.96	-2.36	-1.41	3.15	8.24	6.09	-0.79	5.89	4.66	-2.83	22.17	20.32	12.42	29	27.21	19.49
1	RGU	99.50		0.66	0.21	-0.67	1.39	1.4	0.21	3.71	3.55	3.25	-2.1	0.27	1.73	-0.67	-0.55	0.36	-2.18	-1.8	-1.04	4.03	3.03	1.83	3.54	1.65	1.2	3.77	3.38	2.32	6.07	5.11	3.09
2	RGU	89.40	F2.FT7.T7.PO6.PO8	-4.51	-4.48	-1.16	-8.82	-7.66	-1.43	-3.45	-2.53	-1.49	-0.73	-0.4	0.87	-7.62	-6.34	-1.93	-8.96	-6.4	-0.36	0.86	-1.32	-1.79	-0.9	-2.94	-3.62	15.72	9.32	2.74	19.64	12.16	6.33
3	RGU	97.20		-13.78	-9.27	1.46	-21.12	-15.08	0.96	-5.78	-3.86	4.28	-8.32	-5.25	6.24	-39.12	-24.29	-2.38	-41.35	-28.49	0.01	5.04	4.02	0.84	-8.22	-12.84	-13.43	-6.03	-6.16	-6.68	6.24	2.34	0.3

4	RGU	94.90		-4.54	-2.6	2.05	-8.02	-5.63	0.81	-9.92	-6.85	-0.04	-14.67	-10.76	-0.58	-10.95	-7.33	0.8	-15.04	-11.85	-1.39	6.62	4.9	-2.94	6.4	4.34	-4.22	10.08	9.15	3.38	12.7	11.08	7.46
5	RGU	75.50		-1.73	1.18	5.87	-3.19	-0.78	5.11	-2.01	1.71	7.9	-0.07	2.34	8.23	2.87	4.45	6.01	3.47	6.15	7.12	6.32	1.55	-5.11	2.21	-1.21	-8.32	-3.14	-4.99	-13.48	8.48	3.04	-8.31
6	RGU	68.60																															
7	RGU	79.20		-2.46	0.63	5.83	-1.16	-0.21	8.23	-1.92	-0.68	5.14	2.7	4.67	13.98	-4.33	-3.44	3.68	0.21	-3.41	4.94	-7.16	-4.84	-11.47	-3.85	-2.65	-16.43	7.73	9.53	2.79	12.91	15.65	5.47
8	RGU	92.60		-2.53	1.89	5.42	-2.46	4.01	8.83	-4.32	1.15	4.37	1.88	10.23	11.43	-12.01	-2.66	4.73	-6.58	1.33	4.1	-2.55	-4.59	-7.48	-7.14	-8.27	-9.31	5.73	-1.22	-8.46	13.24	4.71	-1.94
9	RGU	99.50	F8.FC2.M1.O2	-1.81	2.75	8.07	-3.01	0.53	4.43	-4.08	1.02	5.56	-4.8	-0.48	3.06	-5.51	-1.31	4.74	-5.98	-2.88	1.97	3.25	2.41	0.36	1.63	1.89	-1.72	3.4	2.7	-2.81	4.83	4.56	-0.7
10	RGU	96.70		-1.05	-0.93	0.93	-5.9	-4.51	1.42	0.03	0.86	1.86	-8.76	-5.71	-0.03	-2.29	-4.51	-0.7	-5.81	-7.02	0.1	4	2	0.81	0.04	-2.06	-3.03	12.15	8.97	3.02	17.98	12.85	9.16
11	RGU	93.50		5.52	4.22	6.79	3.47	4.23	7.29	6.88	6.13	8.64	10.72	11.54	13.56	3.66	7.24	4.78	-1.42	-1.81	1.56	5.86	6.67	4	-3.24	-1.11	-3.04	6.09	8.99	8.82	12.74	17	17.22
12	RGU	95.80		0.26	3.46	3.86	-1.18	5.13	7.62	-2.65	4.17	5.47	-3.31	5.59	8.56	1.48	5.4	2.9	6.66	10.34	4.63	-0.03	-3.18	-5.15	-2.62	-5.06	-8.77	6.07	-6.55	-13.61	10.8	0.78	-5.02
13	RGU	100.00		1.41	1.13	2.53	0.35	-0.02	0.94	1.96	1.43	-0.4	1.83	2.42	2.15	-25.72	-22.28	-12.28	-14.11	-14.46	-12.15	-9.47	-14.48	-9.45	-20.55	-20.68	-16	-8.14	-13.11	-6.41	4.82	-3.97	5.43
14	RGU	96.30		-10.44	-9.61	-2.3	-17.22	-15.83	-0.99	-6.5	-5.82	1.79	-13.52	-10.61	2.01	-23.44	-20	-3.07	-27.08	-27.43	-11.73	0.09	-2.98	-11.8	-10.67	-10.39	-14.26	28.94	42.92	35.86	44.86	55.1	44.31
15	RGU	98.60		-6.4	-5.42	0.73	-16.05	-14.16	-3.98	-7.08	-5.93	0.49	-12.93	-10.09	-1.1	1.75	2.71	7.63	-5.26	-7.46	-1.04	-12.56	-8.28	-15.72	-15.39	-11.44	-21.31	-10.26	-9.48	-14.15	0.5	0.37	-5.18
16	RGU	100.00	T8.O2	-1.6	-0.08	2.56	-4.05	-2.57	3.79	-1.86	0.13	3.02	-7.94	-4.68	2.83	-4.93	-2.5	1.69	-6.89	-4.81	4.08	-1.72	-4.26	-10.48	-2.84	-4.82	-11.5	6.34	6.86	-0.08	10.03	9.74	2.34
17	RGU	100.00		-0.84	1.67	1.95	-7	-4.27	-1.15	1.23	4.41	6.51	-2.85	1.37	5.4	-11.28	-9.29	-3.99	-9.6	-6.07	-0.62	7.68	6.07	1.24	5.45	4.34	2.71	24.22	18.76	4.56	30.32	23.07	7.95
18	RGU	100.00		-10.19	-10.26	-3.1	-8.52	-4.24	1.88	-7.3	-8.46	-0.62	-3.3	-0.57	3	-13.77	-13.05	-2.2	-14.46	-8.13	1.82	4.64	2.67	-1.84	1.96	0.01	-4.2	9.31	7.53	0.77	13.23	10.18	5.04
19	RGU	59.10																															
20	RGU	74.50																															
21	RGU	100.00		-7.3	-7.23	-3.89	-12	-9.83	-2.91	-8.54	-8.77	-4.36	-9.59	-8.33	-0.81	-11.68	-10.94	-2.66	-11.26	-10.75	-3.68	6.21	5.52	0.55	3.93	3.6	-1.33	0.04	0.63	-3.33	4.26	4.43	-0.09
22	RGU	97.50		-1.3	-0.72	1.05	-3.68	-3.44	-1.21	-3	-5.71	-3.52	-5.02	-6.01	-1.64	-2.78	-2.11	3.06	-5.21	-4.46	1.14	4.93	0.8	-4.96	3.73	-0.75	-6.24	4.71	3.82	-0.58	6.99	5.15	1.1
23	RGU	99.50		-3.39	-2.33	0.07	-8.71	-8.85	-7.98	-10.36	-8.69	-2.1	-8.64	-9.75	-6.76	-5.43	-4.05	-0.76	-4.43	-4.2	-2.9	5.14	4.8	2.79	4.68	4.51	1.53	6.59	4.98	0.5	13.74	12.68	7.8
